# Comparison of tobacco and alcohol use in films produced in Europe, Latin America, and the United States

**DOI:** 10.1186/s12889-015-2378-x

**Published:** 2015-11-03

**Authors:** Inti Barrientos-Gutierrez, Christy Kollath-Cattano, Raul Mejía, Edna Arillo-Santillán, Reiner Hanewinkel, Matthis Morgenstern, James D. Sargent, James F. Thrasher

**Affiliations:** Department of Tobacco Research, National Institute of Public Health (INSP), Universidad No. 655 Colonia Santa María Ahuacatitlán, Cerrada Los Pinos y Caminera C.P, 62100 Cuernavaca, Mor México; Department of Health Promotion, Education & Behavior, Arnold School of Public Health, University of South Carolina, 915 Greene St, 534D, Columbia, SC 29208 USA; Center for Studies of the State and Society (CEDES), Sánchez de Bustamante 27 (C1173AAA) , Buenos Aires, Argentina; Institute for Therapy and Health Research, IFT-Nord, Harmsstrasse 2, 24114 Kiel, Germany; Norris Cotton Cancer Center, Geisel School of Medicine at Dartmouth, 1 Rope Ferry Rd, Hanover, NH 03755 USA

**Keywords:** Media, Global Health, Movies, Tobacco, Alcohol

## Abstract

**Background:**

Studies that have evaluated tobacco and alcohol portrayals in films have mainly focused on US films. Our aim is to describe tobacco and alcohol portrayals in nationally produced films from six European and two Latin American countries, and compare them with US produced films.

**Methods:**

A sample of 337 nationally produced and 502 US produced films, consisting of top grossing films from 2004 to 2009 in each country, was content coded for presence of tobacco or alcohol and seconds of tobacco or alcohol use. Logistic and linear regression models were estimated for all films and youth-rated films (Ages 0–14) to assess cross country differences in tobacco and alcohol content, with US films as the reference category.

**Results:**

Domestically produced films from several countries were more likely than US films to contain any tobacco use both overall (Iceland (OR = 9.29, CI: 1.22–70.89), Italy (OR = 3.58, CI: 1.72–7.43), Argentina (OR = 5.06, CI: 2.13–12.03), Mexico (OR = 4.87, CI: 2.17–10.90)) and for youth-rated films (Germany (OR = 2.24, CI: 1.21–4.16), Iceland (OR = 13.79, CI: 1.80–105.5), Italy (OR = 5.31, CI: 2.54–11.1), and Argentina (OR = 6.9, CI: 0.88–1.34)). Models for alcohol showed few differences compared to US, regardless of rating.

Linear regression models for seconds of use in films with tobacco indicated that only Argentine films had more seconds of smoking than US films, regardless of the rating category. For films with alcohol use, Mexican films had higher seconds of alcohol use than US films.

**Conclusions:**

Smoking was more commonly depicted in films produced outside the US, however there were few differences in the means for smoking screen time in films that contained smoking. This may be partly explained by the prohibition of tobacco product placement in the US. Countries should consider banning paid placement of both products and eliminating subsidies for films with content that promotes tobacco and alcohol use.

## Background

Films are a source of popular entertainment and a powerful promotional vehicle for products and behaviors [1, 2]. Studies in Europe [3–6], the United States (US) [7–9], India [10], and Mexico [11, 12] have found that exposure to onscreen film smoking promotes adolescent smoking. Similar findings have been found for film alcohol portrayals and adolescent drinking in the US [13–15] and Europe [16–18]. Most film exposure to tobacco and alcohol among adolescents in the US, other Western countries, and Latin America comes from Hollywood films, partly because US films dominate theater exhibitions and DVD/Blu-ray sales [19, 20]. In spite of the dominance of Hollywood films in many markets, nationally-produced films may nonetheless meaningfully influence youth smoking and drinking, as some evidence suggests that cultural similarity of youth and film actors enhances the impact of exposure [7, 21]. Furthermore, some policies to reduce youth exposures to film drinking and smoking, such as prohibiting government subsidies for films with tobacco [22], are more likely to influence nationally-produced films than foreign films. Because nationally-produced films may both have a greater effect on youth risk behaviors and be easier to regulate, it is important to monitor their content.

The only study to assess the impact of non-US, nationally-produced films (i.e. Bollywood) found an association between exposure to film smoking and smoking behavior among Indian adolescents [10]. Studies in Europe [5, 17] and Mexico [11, 12] have assessed the effects of youth exposure to tobacco imagery in both US- and nationally-produced films; however, these studies have not reported on the extent of alcohol or tobacco content in nationally-produced films. Only three other studies of which we are aware analyzed the content of non-US films, describing alcohol and drug use portrayals in Brazilian films [23], alcohol brand appearances in UK produced films [24], and substance use portrayals in Nigerian films [25]. Further research on nationally-produced films is necessary to inform policy actions to limit youth exposures to film portrayals of tobacco and alcohol, including policies prohibiting brand appearances and subsidies that the World Health Organization (WHO) recommends [22] and that would have a greater influence on national film production than on the production of imported films, including Hollywood films.

The current study aimed to describe and compare tobacco and alcohol portrayals in films released from 2004 to 2009 and produced in the US, six European countries (i.e., Germany, Iceland, Italy, Netherlands, Poland, UK) and two Latin American countries (i.e., Mexico, Argentina). We hypothesized that US-produced films would be less likely to contain tobacco than films from other countries, because paid tobacco product placement in Hollywood films was prohibited in 1997 and because film portrayals of tobacco use declined over the observation period [26, 27]. Prohibitions against tobacco product placement in other countries were mostly implemented more recently. Because we know of no product placement restrictions for alcohol, we had no expectations about differences across countries.

## Methods

This study incorporated data from the following three projects: Smokefree Movies Europe [5], Cinema and Youth Smoking in Latin America, Dartmouth Media Research Laboratory ongoing media content analysis project [26]. All three projects included an analysis of films released between 2004 and 2009 and that were commercially successful in their respective countries. Successful European films (*n* = 220) were determined by box office data on the 50 most successful films within each country from 2005, 2006, 2007, and 2008, and the 25 most successful films from 2004 and 2009. Mexican and Argentine films (*n* = 117) were selected from the 100 most successful films from each year from 2004 to 2009. Nationally produced films were defined as films that were mainly produced by the country in which box office data were collected. A comparison sample of US-produced films (*n* = 502) consisted of films that were already coded by the Dartmouth Media Research Laboratory and were commercially successful from the time period of 2004 to 2009 in at least one of the eight comparison countries, according to the same box office data used to select nationally produced films.

**Fig. 1:**
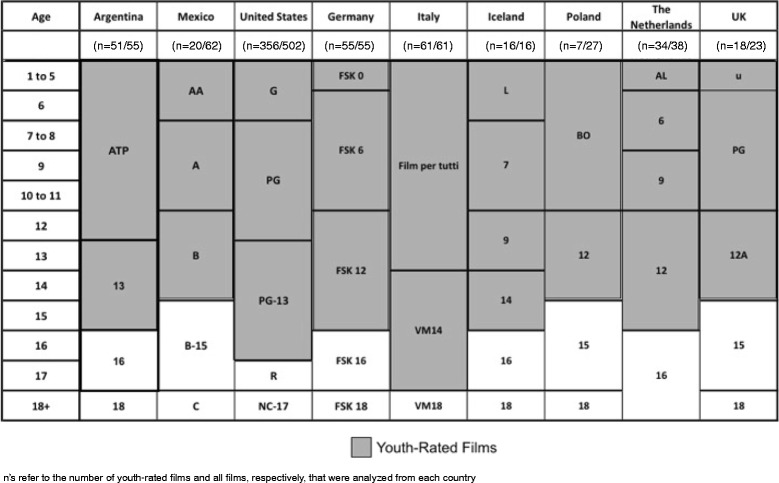
Film rating categories used in study countries

Film ratings were found through the Internet Film Database for European and US films, and through government entities in Argentina (i.e., Instituto Nacional de Cine y Artes Audiovisuales) and Mexico (i.e., Dirección General de Radio, Televisión, y Cinematografía). Rating systems use age thresholds that vary across countries (Fig. 1), and a rating for ages 0–14 was created for cross-country comparisons of “youth-rated” films.

Film content coding was based on a validated system [28]. Since the study incorporated data from three different projects, each project used slightly different methods for coding films. Tobacco and alcohol in European films (*n* = 220) was coded by occurrence, where an occurrence was recorded every time there was use or implied use of tobacco or alcohol. For Latin American and US films, tobacco and alcohol were coded based on seconds of appearance on screen. However, for analytic purposes European occurrences were transformed into seconds using a linear regression equation with slope and intercept values obtained from models that regressed tobacco or alcohol occurrences on tobacco or alcohol seconds for the same 20 films. In order to determine inter-rater reliability, a small percentage of films per country (10 % US, 20 % Latin America, 20 % Europe) were double coded (r = 0.92–0.99 for tobacco; r = 0.93–0.99 for alcohol).

### Analysis

Data were analyzed using STATA version 13 [29]. The prevalence of films with any tobacco or alcohol use was calculated for all films, then for youth-rated films in each country, because WHO recommends using youth ratings to reduce youth exposures to risk behaviors in films [22]. For films that contained alcohol and/or tobacco, means of alcohol and/or tobacco seconds were assessed. Logistic (prevalence) and linear (seconds) regression models were estimated separately for alcohol and tobacco for all films and for youth-rated films, using dummy variables for country of film production with US-produced films as the reference group.

Since this study was based on a secondary analysis of data from three different projects and data were manipulated for analytic purposes, a sensitivity analysis that utilized original data was conducted. Separate linear regressions models were estimated for 1) occurrences for European countries, using the UK as the comparison group; and 2) seconds for Latin American countries using US films as the comparison group. The UK was chosen as the comparison group for European countries due to the similarities in percentages of films with tobacco and alcohol between the US and the UK and the fact that many nationally produced UK films were co-produced with US companies. The main models that included seconds for all nine countries were also estimated using the UK as a comparison group.

## Results

### Tobacco

**Fig. 2:**
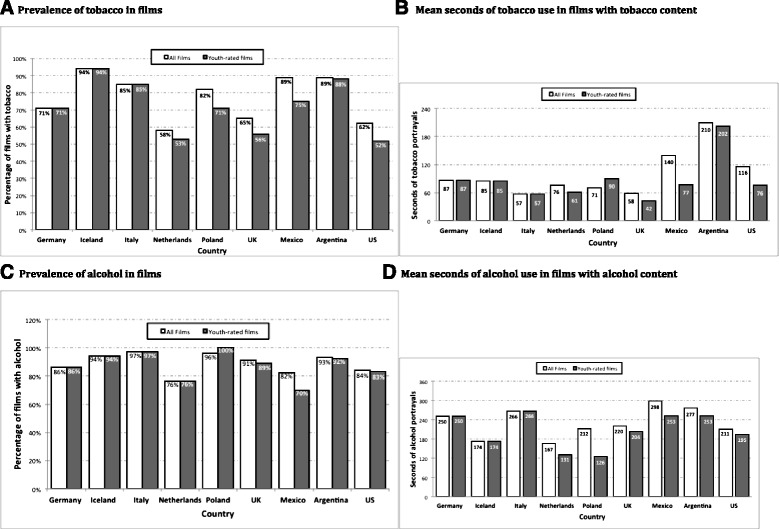
**a**-**d** Prevalence and duration of tobacco and alcohol use in films produced in Europe, Latin America, and the US

When comparing the percentage of films with tobacco across all countries, the Netherlands had the lowest percentage of films with tobacco (58 % of all films; 53 % of youth-rated films) and Icelandic films had the highest (94 % of all films, all youth rated). Of US-produced films, 62 % contained tobacco, with 52 % of youth-rated films containing tobacco (Fig. 2a). In logistic regression models with all films, films from Iceland, Italy, Argentina, and Mexico were more likely to contain tobacco than US films. In logistic regression models with only youth-rated films, films from Germany, Iceland, Italy, and Argentina were more likely to contain tobacco than US films (Table 1).

**Table 1:** Logistic and linear regression models assessing differences in tobacco portrayals between US and nationally produced films

	Tobacco							
	Youth-rated films				All films			
	Prevalence		Seconds		Prevalence		Seconds	
Country	OR	95 % CI	β	95 % CI	OR	95 % CI	β	95 % CI
US	ref.		ref.		ref.		ref.	
Germany	2.24*	(1.21–4.16)	10.86	(−38.25–59.98)	1.51	(0.82–2.78)	−28.68	(−85.65–28.29)
Iceland	13.79*	(1.80–105.50)	9.48	(−65.38–84.33)	9.29*	(1.22–70.89)	−30.06	(−118.71–58.58)
Italy	5.31***	(2.54–11.10)	−19.36	(−63.11–24.38)	3.58**	(1.72–7.43)	−58.90*	(−109.15–(−)8.65)
Netherlands	1.03	(0.51–2.09)	−14.74	(−83.58–54.10)	0.85	(0.44–1.66)	−39.51	(−113.50–34.47)
Poland	2.30	(0.44–2.98)	14.23	(−112.15–140.62)	2.72	(1.02–7.32)	−44.78	(−118.76–29.20)
UK	1.15	(0.98–7.75)	−34.09	(−124.62–56.44)	1.16	(0.48–2.79)	−57.67	(−146.32–30.97)
Mexico	2.76	(2.87–16.57)	0.75	(−74.10–75.61)	4.87***	(2.17–10.90)	24.83	(−24.23–73.89)
Argentina	6.90***	(0.88–1.34)	126.27***	(79.94–172.60)	5.06***	(2.13–12.03)	94.50***	(43.96–146.05)

*p < .05, **p < .01, ***p < .001

Mean seconds of tobacco use in movies that contained tobacco were compared for all countries. Linear regression models indicated that only Argentine films had significantly more seconds of smoking than US films for both all films and youth-rated films (Table 1). The means were 210 (all films) and 202 (youth-rated films) for Argentina compared to 116 (all films) and 76 (youth-rated films) for the US (Fig. 2b). On the other hand Italy had significantly less seconds of smoking than US films for all films (Table 1).

### Alcohol

The prevalence of films containing alcohol was generally higher and less variable across countries than for tobacco (range = 76 % in Netherlands to 97 % in Italy, with US = 84 %, see Fig. 2c). Similarly high percentages were found in youth-rated films (range = 70 % in Mexico to 100 % in Poland, with US = 83 %). In logistic models predicting the presence of alcohol, whether considering all films or youth-rated films, only Italian films were more likely to contain alcohol than US films (Table 2).

**Table 2:** Logistic and linear regression models assessing differences in alcohol portrayals between US and nationally produced films

	Alcohol							
	Youth-rated films				All films			
	Prevalence		Seconds		Prevalence		Seconds	
Country	OR	95 % CI	β	95 % CI	OR	95 % CI	β	95 % CI
US	ref.		ref.		ref.		ref.	
Germany	1.28	(0.58–2.85)	55.11	(−8.87–119.08)	1.11	(0.51–2.45)	38.89	(−30.76–108.54)
Iceland	3.28	(0.43–25.25)	−20.28	(−128.07–87.51)	2.84	(0.37–21.83)	−36.50	(−155.50–82.51)
Italy	6.44*	(1.53–27.06)	70.93*	(12.83–129.03)	5.59*	(1.34–23.35)	54.71	(−8.24–117.66)
Netherlands	0.71	(0.31–1.64)	−63.63	(−146.95–19.69)	0.61	(0.28–1.34)	−44.04	(−130.98–42.91)
Poland	omitted^a^		−68.95	(−224.67–86.77)	4.93	(0.46–8.66)	0.66	(−90.86–92.18)
UK	1.75	(0.39–7.79)	9.73	(−94.80–114.27)	1.99	(0.44–1.76)	9.51	(−91.75–110.76)
Mexico	0.51	(0.19–1.38)	58.54	(−53.10–169.67)	0.87	(0.85–6.88)	87.40*	(20.26–154.54)
Argentina	2.57	(0.89–7.38)	58.54	(−5.44–122.52)	2.42	(4.15–6.70)	65.75	(−1.39–132.90)

*p < .05, **p < .01, ***p < .001

Note^a^: Omitted due to predicting success perfectly (100 % of films contain alcohol)

Screen-time alcohol imagery in films that contained alcohol ranged from 211 s in US films to 298 s in Mexican films (Fig. 2d). Linear regression models for all films indicated that only Mexican films had higher screen time alcohol presence than US films (Table 2). For youth-rated films seconds of alcohol screen time ranged from 126 in Polish films to 266 in Italian films. Linear regression models indicated that youth-rated Italian films contained more alcohol content than US films.

### Sensitivity analysis

The sensitivity analysis yielded similar results and found no differences between European countries in number of tobacco or alcohol occurrences for all films or youth-rated films. Models that only considered seconds of tobacco or alcohol use for Latin American and US films yielded the same results as for models that included seconds for all nine countries.

## Discussion

This study finds that alcohol and tobacco are present in most films, regardless of the country of production. Results were similar when examining only youth-rated films. Moreover, youth-rated films appear just as likely to contain alcohol as all films produced in about half of the countries examined, partly because most nationally-produced films receive youth ratings. If rating films for youth increases the likelihood of youth exposure to alcohol and tobacco portrayals, then countries should consider their inclusion in rating systems, as recommended by the WHO to prevent youth tobacco use [22].

Policies that prohibit product placement may help explain differences in content across nationally-produced films. For example, relatively lower levels of tobacco in US films are likely due to the 1997 prohibition of tobacco product placement, after which tobacco portrayals in US films declined [27, 30]. The European Union’s (EU) 2003 Tobacco Advertising Directive prohibited tobacco product placement in films, which EU countries should have adopted into national legislation by 2005 [31]. The Netherlands, the UK, and Poland had all adopted these policies by 2005, which may explain why the prevalence of tobacco was not significantly different from US-produced films. On the other hand, Italy and Germany were referred to the European Court of Justice for not adopting this measure. In 2008, Italy had implemented a ban but Germany had not [32], although the Italian ban is unlikely to have influenced films considered here (i.e. released 2004–2009) because of the delay between film shooting and screening. As a non-EU member, Iceland was not required to, nor did it, prohibit tobacco product placement in films during the study period. The same applies to Mexico and Argentina. Our study found that films produced in countries without policies prohibiting tobacco product placement were more likely to include tobacco than US films, suggesting that these policies have influenced film production.

Even in countries where tobacco product placement policies have been implemented, more than half of films contain tobacco. To further decease tobacco portrayals and its consequences, other measures that the WHO recommends may be necessary, including giving adult ratings to films with tobacco, certification of no payoffs from the tobacco industry, and prohibition of tobacco in government subsidized films [22]. Because nationally-produced films are often subsidized by the government [22], this measure may be particularly effective for influencing locally produced films. Indeed, policy makers are more likely to influence nationally-produced films than foreign films.

There is no clear country-specific pattern to the data on alcohol, although its presence is universally very high, albeit highest in Italy. Even though exposure to alcohol imagery through entertainment media appears to promote youth alcohol use [13–18], to our knowledge, no country considered here has implemented policies to reduce alcohol use in films. The WHO encourages prohibition of alcohol marketing in cultural activities, sponsorships and product placement, including new marketing strategies (e-mails, SMS, podcasting, social media) [33]. Some European countries have restricted promotion of alcoholic beverages [34] but policies to restrict alcohol product placement in movies has not and should be considered. However, the case of tobacco indicates that additional policies, like those recommended for tobacco, may be necessary to substantially reduce alcohol content in films. Nevertheless, countries have been slow to adopt WHO-recommended policies for tobacco, in spite of tobacco’s detrimental health impact at any level of exposure and its devastating public health impact. Such policies may be even more difficult to advance in the domain of alcohol because of widespread beliefs that alcohol consumption can be safe and even healthful at moderate levels. However, the scientific data that supports these beliefs has been questioned [35–39], and the negative social and public health impacts of excessive alcohol use are clear. To be successful, global public health stakeholders, such as the WHO, and civil society organizations will likely need to organize around this issue to provide clear recommendations like those advanced for tobacco, while also anticipating counterarguments and resistance against these policies [40].

This study has several limitations, including not examining the context of tobacco or alcohol use and the small number of nationally-produced films for some countries. Because US films dominate the markets considered in this study, they likely account for a higher percentage of tobacco and alcohol exposures amongst youth, even though US-produced films are less likely than nationally-produced films to include tobacco use. Further research is necessary to determine relative contribution and impact of substance use portrayals in entertainment media from national- compared to foreign-produced films, as well as from different media modalities (e.g., television, Internet, films). These and other potential weaknesses of the current study are counterbalanced by the very large sample of films from multiple, diverse countries that we analyzed. To our knowledge, this is largest international study of substance use in nationally-produced films. Future research should provide a more nuanced portrait of the context of substance use across countries and whether the effects on youth risk taking differ by context of use or country of production.

## Conclusions

Smoking and drinking were highly prevalent in nationally-produced films, however for many countries only the percentage of nationally-produced films that contained tobacco was consistently different than the percentage of US-produced films. Nationally produced films may exert an important impact if the cultural similarity of film characters and audiences enhances their effects, as has been found for some studies [7, 21]. To reduce such effects, countries could consider WHO-recommended policies of prohibiting tobacco use in films that receive government subsidies [22], as these films often represent the majority, if not all, of nationally-produced films. Future research should examine the effects of tobacco and alcohol portrayals in films, as well as the efficacy of policies that aim to reduce youth exposures to these portrayals.
